# The Inhibitory Effect of α/β-Hydrolase Domain-Containing 6 (ABHD6) on the Surface Targeting of GluA2- and GluA3-Containing AMPA Receptors

**DOI:** 10.3389/fnmol.2017.00055

**Published:** 2017-03-02

**Authors:** Mengping Wei, Moye Jia, Jian Zhang, Lulu Yu, Yunzhi Zhao, Yingqi Chen, Yimeng Ma, Wei Zhang, Yun S. Shi, Chen Zhang

**Affiliations:** ^1^State Key Laboratory of Membrane Biology, School of Life Sciences, Peking UniversityBeijing, China; ^2^PKU-IDG (International Digital Group)/McGovern Institute for Brain Research, Peking UniversityBeijing, China; ^3^Department of Pharmacology, Institute of Chinese Integrative Medicine, Hebei Medical UniversityShijiazhuang, China; ^4^Ministry of Education (MOE) Key Laboratory of Model Animal for Disease Study, Model Animal Research Center of Nanjing UniversityNanjing, China

**Keywords:** AMPA receptor, ABHD6, receptor trafficking, glutamates, protein–protein interactions

## Abstract

The α-amino-3-hydroxy-5-methyl-4-isoxazolepropionic acid (AMPA)-type glutamate receptors (AMPARs) are major excitatory receptors that mediate fast neurotransmission in the mammalian brain. The surface expression of functional AMPARs is crucial for synaptic transmission and plasticity. AMPAR auxiliary subunits control the biosynthesis, membrane trafficking, and synaptic targeting of AMPARs. Our previous report showed that α/β-hydrolase domain-containing 6 (ABHD6), an auxiliary subunit for AMPARs, suppresses the membrane delivery and function of GluA1-containing receptors in both heterologous cells and neurons. However, it remained unclear whether ABHD6 affects the membrane trafficking of glutamate receptor subunits, GluA2 and GluA3. Here, we examine the effects of ABHD6 overexpression in HEK293T cells expressing GluA1, GluA2, GluA3, and stargazin, either alone or in combination. The results show that ABHD6 suppresses the glutamate-induced currents and the membrane expression of AMPARs when expressing GluA2 or GluA3 in the HEK293T cells. We generated a series of GluA2 and GluA3 C-terminal deletion constructs and confirm that the C-terminus of GluAs is required for ABHD6’s inhibitory effects on glutamate-induced currents and surface expression of GluAs. Meanwhile, our pull-down experiments reveal that ABHD6 binds to GluA1–3, and deletion of the C-terminal domain of GluAs abolishes this binding. These findings demonstrate that ABHD6 inhibits the AMPAR-mediated currents and its surface expression, independent of the type of AMPAR subunits, and this inhibitor’s effects are mediated through the binding with the GluAs C-terminal regions.

## Introduction

In the mammalian brain, α-amino-3-hydroxy-5-methyl-4-isoxazolepropionic acid (AMPA)-type glutamate receptors (AMPARs) are major ionotropic receptors that mediate the majority of fast excitatory synaptic transmissions. The binding of the glutamate released from presynaptic terminals with postsynaptic AMPARs determines the efficiency and plasticity of synaptic transmission between pairs of neurons. The numbers and biophysical properties of AMPARs remain dynamically modulated during periods of rest and plasticity, and deficits in these processes are strongly linked to psychiatric and neurodegenerative diseases, including Huntington’s disease and Alzheimer’s disease (Keinänen et al., 1990; Jackson and Nicoll, 2011; Huganir and Nicoll, 2013; Henley and Wilkinson, 2016).

AMPARs are tetramers assembled from four AMPAR subunits: GluA1–4 (Hollmann and Heinemann, 1994). The subunit composition determines the biophysical and molecular properties of AMPARs. The C-terminus of AMPARs interacts with proteins, such as PICK1, NSF, GRIP1, SAP97, and NSF, to traffic the receptors to the plasma membrane and the synapse in both constitute and activity-dependent manners (see reviews Ziff, 2007; Anggono and Huganir, 2012; Cheng et al., 2012; Yang et al., 2014). For example, PICK1 causes a selective decrease in the surface expression levels of GluA2, while the surface expression levels of GluA1 remain unchanged, suggesting that PICK1 regulates the subunit composition and synaptic targeting of surface AMPARs (Terashima et al., 2004; Cao et al., 2007; Xu et al., 2016). GluA2 confers the calcium permeability to the AMPARs; while GluA2-containing receptors are not calcium permeable, GluA1-containing receptors are calcium permeable. All AMPAR subunits are subject to alternative splicing to generate either “flop” or “flip” versions of the receptors, while only GluA2 undergoes Q/R RNA editing (Burnashev et al., 1992).

The gating, pharmacology, trafficking, and localization of AMPARs are regulated by not only the subunit composition of GluA subunits, but also the AMPARs auxiliary subunits proteins (Bredt and Nicholl, 2003; Li et al., 2016). Stargazin was identified as the first member in the family of transmembrane AMPAR regulatory proteins (TARPs; Letts et al., 1998; Chen et al., 1999, 2000; Hashimoto et al., 1999), and ever since, more and more AMPAR-associated proteins are identified, mainly using proteomics approaches. Native AMPARs associate with a variety of regulatory proteins, including TARPs, cornichon-2 and -3, cystine-knot AMPAR modulating protein of 44 kDa (CKAMP44), germ cell-specific gene 1-like protein (GSG1L), α/β-hydrolase domain-containing 6 (ABHD6), porcupine (PORCN), etc. (Vandenberghe et al., 2005; Tomita et al., 2006; Milstein and Nicoll, 2008; Coombs et al., 2012; Schwenk et al., 2012; Gu et al., 2016b; Wei et al., 2016). The functional analysis of these proteins reveals the AMPARs’ important and distinct roles in both neurons and heterologous cells. For example, type I TARPs (stargazin, γ-3, γ-4, γ-8) are necessary and sufficient for the delivery of AMPARs to the plasma membrane in cerebellum granule cells. Furthermore, type I TARPs also modulate the properties of the AMPAR channels by reducing desensitization and slowing the deactivation of the AMPARs (Priel et al., 2005; Tomita et al., 2006). GSG1L and ABHD6 suppress AMPAR-mediated synaptic transmission and modulate its kinetics in hippocampal neurons (Gu et al., 2016a; Wei et al., 2016). Cornichon-2 and -3 conditional knock-out mice showed selective reduction of surface GluA1-containing subunits, and impaired strength and kinetics of AMPAR-mediated synaptic transmission. This is likely due to the effect of TARPγ-8, which can mediate the functional interaction between CNIHs and AMPARs, thus promoting the association of CNIHs with the GluA1 subunit and preventing the association of CNIHs with other subunits (Herring et al., 2013).

We previously showed that ABHD6, a monoacylglycerol lipase, can bind to the C-terminus of GluA1. Overexpression of ABHD6 can reduce AMPAR-mediated excitatory neurotransmission in neurons and glutamate-induced currents in HEK293T cells in a 2-arachidonoylglycerol independent manner. Further studies via immunostaining demonstrated that these decreases might have been due to the decreased surface expression levels of GluA1, rather than the overall expression levels (Wei et al., 2016). Thus, ABHD6 seems to functionally interact with GluA1 in both heterologous cells and cultured hippocampal neurons. However, whether the inhibitory effect of ABHD6 on AMPAR function depends on the type of AMPAR subunit remains uncertain. In the present study, we investigated the subunit specificity of ABHD6’s inhibition on AMPARs in transfected HEK293T cells.

## Materials and Methods

### Construction of Expression Vectors

Rat GluA1, GluA2, and GluA3 subunit cDNAs containing internal ribosome entry site linked green fluorescent protein (IRES-GFP) were used in the present study and have been described previously (Shi et al., 2009). ABHD6-2A-GFP was cloned into a pFUGW expression vector using polymerase chain reaction (PCR) methods (Wei et al., 2016). GluA1, GluA2, and GluA3 deletion constructs were generated by PCR. GluA1-deletion 14 (A1D14) ended in SKRMK; GluA2-deletion 1 (A2D1) ended in EGYNV; GluA2-deletion 2 (A2D2) ended in QNSQN; GluA2-deletion 3 (A2D3) ended in SQNSQ; GluA2-deletion 4 (A2D4) ended in SSQNS; GluA2-deletion 5 (A2D5) ended in SSSQN; GluA2-deletion 6 (A2D6) ended in PSSSQ; GluA2-deletion 7 (A2D7) ended in NPSSS; GluA2-deletion 8 (A2D8) ended in KNPQN; GluA2-deletion 9 (A2D9) ended in RMKVA; GluA2-deletion 10 (A2D10) ended in KRMKV; GluA2-deletion 11 (A2D11) ended in AKRMK; GluA2-deletion 12 (A2D12) ended in EAKRM; GluA3-deletion 1 (A3D1) ended in NTQNY; GluA3-deletion 2 (A3D2) ended in KPAPA; GluA3-deletion 3 (A3D3) ended in FKPAP; GluA3-deletion 4 (A3D4) ended in NFKPA; GluA3-deletion 5 (A3D5) ended in KNTQN; GluA3-deletion 6 (A3D6) ended in RMKLT; GluA3-deletion 7 (A3D7) ended in KRMKL; and GluA3-deletion 8 (A3D8) ended in SKRMK. All the C-terminal deletion constructs were tagged with an human influenza hemagglutinin (HA) tag with a linker of GQG (**Figure 3A**). GluA1ΔATD lacked sequence from ANFPN to DDKFV, GluA2ΔATD lacked sequence from VSSNS to VDKMV, and GluA3ΔATD lacked sequence from GFPNT to YERFV. All the N-terminal deletion constructs were tagged with a Flag tag (**Figure 4A**). Myc-ABHD6 was cloned into a pCAG vector using PCR methods. The constructs were verified with Sanger sequencing.

### HEK293T Cell Culture and Transfection

HEK293T cells (KCB Cat# KCB 200744YJ, RRID:CVCL_0063) were used for expressing GluAs, stargazin, control, and ABHD6. The HEK293T cells were cultured with 5% CO_2_ in a 37°C incubator (Jiang et al., 2015). The cDNA transfection was performed in 3.5-cm dishes or six-well plates. The total cDNA used for transfection per 3.5-cm dish or per well in six-well plates was 4 μg. When the expression was performed, a 2:3 ratio of GluA to stargazin cDNA was used (we increased the GluA3 to stargazin ratio to 4:1 to get detectable currents). When GluA1 and GluA2 were coexpressed, the ratio of GluA1 to GluA2 was 3:2 (Shi et al., 2009). Transfection was terminated after 3–5 h. All of the HEK293T cell transfections were performed using polyethylenimine (Polysciences, USA). The HEK293T cells were dissociated with 0.05% trypsin and plated on poly-D-lysine-pretreated coverslips after counting the cells with an automated cell counter (μScope CellCounter Basic, Zhoushan Chengchuang Electronic Tech. Co., China). Electrophysiological recording was performed 24–48 h after transfection.

### Electrophysiological Recordings

Electrophysiological recordings were performed as previously reported (Zhang et al., 2009, 2010). Whole-cell voltage clamp recordings were performed for HEK293T cells with a MultiClamp 700A amplifier (Molecular Devices). Series resistance was compensated to 60–70%, and recordings with series resistances of >20 MΩ were rejected. The data were analyzed using Clampfit 9.02 (pClamp, RRID:SCR_011323), Igor 4.0 (WaveMetrics), and Prism 5 (GraphPad Prism, RRID:SCR_002798). Data were presented as mean ± SEM. Differences in means were tested with Student’s *t*-test and were accepted as significant if *p* < 0.05. Coverslips with transfected HEK293T cells were maintained during the recordings in an external solution containing (in mM) NaCl 144, KCl 10, CaCl_2_ 2, MgCl_2_ 1, HEPES 10, and D-glucose 10, with the pH adjusted to 7.4, mOsm/kg 315. Using 3–5 MΩ borosilicate glass pipettes (World Precision Instruments), whole-cell patches were excised from positively transfected cells identified by epifluorescence microscopy. The internal solution contained (in mM) KCl 145, NaCl 5, EGTA 5, MgATP 4, Na_2_GTP 0.3, and HEPES 10, with the pH adjusted to 7.2, Osm 305. The glutamate-induced currents were recorded by the local puffing of bath solution containing potassium glutamate (10 mM), and the cells were background perfused with bath solution at the speed of 3 mL/min.

### Immunostaining Analyses

Immunofluorescence analyses were performed as previously described. Experiments were performed under non-permeabilized conditions to label the surface of the GluAs receptors. The coverslips with transfected HEK293T cells were washed once with phosphate-buffered saline (PBS). The PBS solution contained (g/L) NaH_2_PO_4_⋅H_2_O 3.1, Na_2_HPO_4_ 10.9 and NaCl 9, with the pH adjusted to 7.4, Osm 310, fixed with 4% formaldehyde in PBS for 12 min at room temperature (RT), washed three times with PBS, and then blocked with PBS containing 3% goat serum and 5% milk for 30 min at RT. The cells were then incubated for 2 h at RT with the primary antibody (Millipore Cat# AB1504 RRID:AB_2113602; HA 1:1000 Abmart) diluted in a blocking solution. Either a donkey anti-rabbit Alexa Fluor 546-conjugated secondary antibody (Life Technologies) or a goat anti-mouse Alexa Fluor 633-conjugated secondary antibody (Life Technologies) was used at 1:500 according to the source of primary antibody. The cells were incubated for 1 h at RT with the secondary antibody and washed three to five times with PBS. To label the total GluAs, 0.2% triton was used 5 min after fixation. Fluoromount-G (Southern Biotech) was used to mount the cells on microscope slides. Images were acquired with a laser scanning confocal microscope (Olympus), and were further analyzed in a blinded fashion using the National Institutes of Health (NIH) ImageJ program (ImageJ, RRID:SCR_003070).

### Affinity Chromatography Experiments and Western Blotting

To examine the binding region of GluAs with ABHD6 in HEK293T cells, 7.5 μg full-length GluAs or GluAs deletion plasmids, together with 2.5 μg ABHD6, were transfected in a 60-mm dish. A pCAG empty vector was used as control. The cells were harvested 48 h after transfection. The HEK293T cells were washed with PBS once, kept at -80°C overnight, and thawed at 37°C for 1 min. Then, the cells were collected with PBS and centrifuged at 17,000× *g* for 1 min at 4°C to obtain the cell pellets. 200 μl of buffer A (150 mM NaCl, 20 mM HEPES, 2 mM CaCl_2_, 2 mM MgCl_2_, 0.1 mM EDTA, 1% Triton, and protease inhibitors) was added to the cell pellets. Proteins were solubilized by gentle rocking at 4°C for 2 h. Next, the insoluble fractions were removed by centrifugation at 17,000× *g* for 30 min. A total of 150 μl of supernatant was used for an affinity chromatography assay, and 16 μl were used as inputs. 3 μl anti-myc antibodies (M20002, Abmart) and 24 μl of protein G beads were added to samples and rotated overnight at 4°C. Then, the beads were washed five times with wash buffer (150 mM NaCl, 20 mM HEPES, 2 mM CaCl_2_, 2 mM MgCl_2_, 0.1 mM EDTA, 1% Triton, and protease inhibitors), boiled in SDS sample buffer, and subjected to sodium dodecyl sulfate polyacrylamide gel (SDS-PAGE) electrophoresis.

### Western Blotting

SDS-PAGE was performed using NuPAGE precast gels (10% Bis-Tris gels, Life Technology), then transferred to nitrocellulose (HATF00010, Millipore), and visualized on immunoblots. The bounded secondary antibody (IRDye^®^ 680LT Goat anti-Mouse IgG and 800CW Goat anti-Rabbit IgG, Odyssey) was detected by an infrared imaging system (Odyssey). Monoclonal antibodies against HA-tag (M20003; Abmart) and polyclonal antibodies against GluA1 (ab1504; Millipore) were used in this study.

## Results

### Overexpression of ABHD6 Decreased GluA2- or GluA3-Mediated Currents in Transfected HEK293T Cells

**FIGURE 1:**
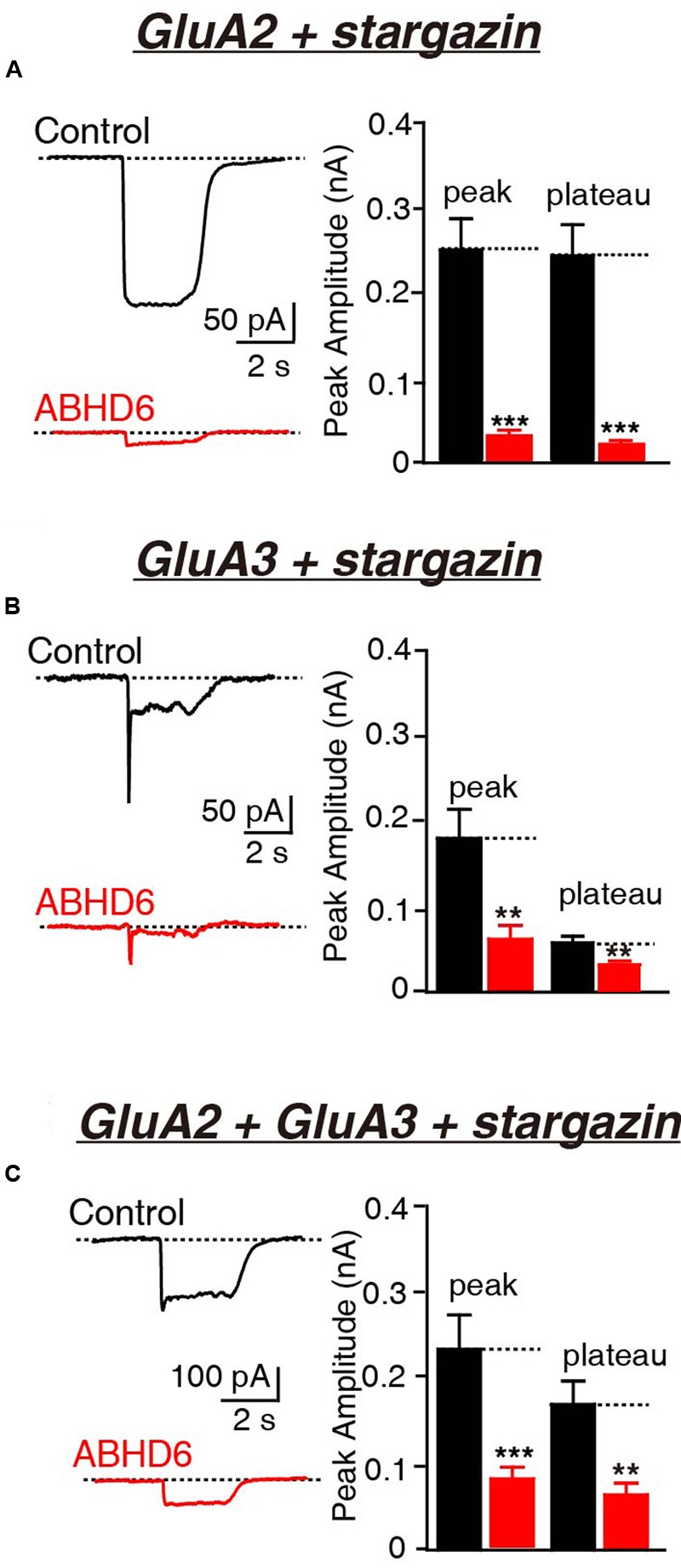
**Overexpression of ABHD6 decreased GluA2- or GluA3-mediated currents in HEK293T cells co-transfected with stargazin.** All data are from HEK293T cells transfected with either an empty vector (control) or a vector encoding ABHD6-2A-GFP in electrophysiological experiments **(A–C)**. **(A)** Representative traces (left) and summary graphs of the peak amplitudes and plateaus (right) of 10 mM glutamate-induced currents in HEK293T cells transfected with combinations of GluA2 and stargazin (control: *n* = 30/3; ABHD6: *n* = 30/3; peak: *p* < 0.0001; plateau: *p* < 0.0001). **(B)** Representative traces (left) and summary graphs of the peak amplitudes and plateaus (right) of 10 mM glutamate-induced currents in HEK293T cells transfected with combinations of GluA3 and stargazin (control: *n* = 27/3; ABHD6: *n* = 27/3; peak: *p* < 0.01; plateau: *p* < 0.01). **(C)** Representative traces (left) and summary graphs of the peak amplitudes and plateaus (right) of 10 mM glutamate-induced currents in HEK293T cells transfected with combinations of GluA2, GluA3, and stargazin (control: *n* = 24/3; ABHD6: *n* = 24/3; peak: *p* < 0.001; plateau: *p* < 0.01). All summary graphs show means ± SEMs; statistical comparisons by Student’s *t*-test yielded: ^∗^*p* < 0.05, ^∗∗^*p* < 0.01, ^∗∗∗^*p* < 0.001.

**FIGURE 2:**
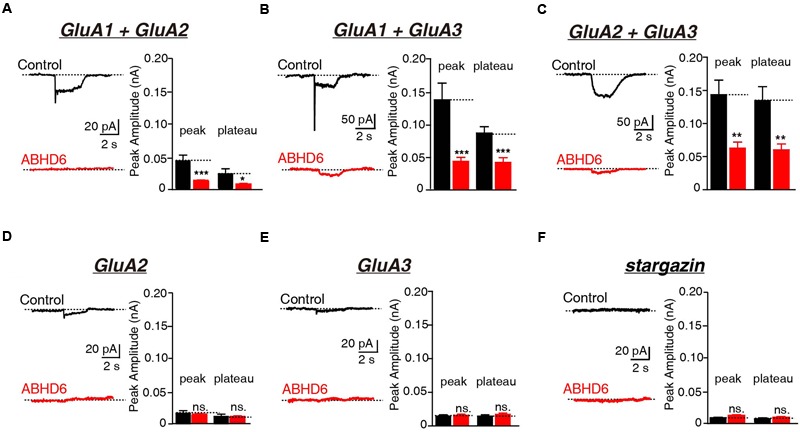
**Overexpression of ABHD6 decreased GluA2- or GluA3-mediated currents in transfected HEK293T cells without stargazin. (A)** Representative traces (left) and summary graphs of the peak amplitudes and plateaus (right) of 10 mM glutamate-induced currents in HEK293T cells transfected with combinations of GluA1 and GluA2 (control: *n* = 24/3; ABHD6: *n* = 24/3; peak: *p* < 0.001; plateau: *p* < 0.05). **(B)** Representative traces (left) and summary graphs of the peak amplitudes and plateaus (right) of 10 mM glutamate-induced currents in HEK293T cells transfected with combinations of GluA1 and GluA3 (control: *n* = 23/3; ABHD6: *n* = 23/3; peak: *p* < 0.001; plateau: *p* < 0.001). **(C)** Representative traces (left) and summary graphs of the peak amplitudes and plateaus (right) of 10 mM glutamate-induced currents in HEK293T cells transfected with combinations of GluA2 and GluA3 (control: *n* = 24/3; ABHD6: *n* = 23/3; peak: *p* < 0.01; plateau: *p* < 0.01). **(D)** Representative traces (left) and summary graphs of the peak amplitudes and plateaus (right) of 10 mM glutamate-induced currents in HEK293T cells transfected with GluA2 (control: *n* = 18/3; ABHD6: *n* = 18/3; peak: *p* > 0.05; plateau: *p* > 0.05). **(E)** Representative traces (left) and summary graphs of the peak amplitudes and plateaus (right) of 10 mM glutamate-induced currents in HEK293T cells transfected with GluA3 (control: *n* = 18/3; ABHD6: *n* = 18/3; peak: *p* > 0.05; plateau: *p* > 0.05). **(F)** Representative traces (left) and summary graphs of the peak amplitudes and plateaus (right) of 10 mM glutamate-induced currents in HEK293T cells transfected with stargazin (control: *n* = 18/3; ABHD6: *n* = 18/3; peak: *p* > 0.05; plateau: *p* > 0.05). All summary graphs show means ± SEMs; statistical comparisons by Student’s *t*-test yielded: ^∗^*p* < 0.05, ^∗∗^*p* < 0.01, ^∗∗∗^*p* < 0.001.

Previously, we demonstrated that the overexpression of ABHD6 suppressed the glutamate-induced current in HEK293T cells expressing GluA1, GluA1 + stargazin, and GluA1 + GluA2 + stargazin (Wei et al., 2016). Since GluA1–3 are the major AMPAR subunits expressed in the brain (Angulo et al., 1997; Lee et al., 1998; Lu et al., 2009), we focused our analysis on GluA2 and GluA3, either alone or in combination. The overexpression of ABHD6 significantly reduced the peak amplitude and steady-state amplitude of glutamate-induced currents in HEK293T cells expressing GluA2 (**Figure 1A**), GluA3 (**Figure 1B**), or GluA2 + GluA3 (**Figure 1C**), when co-transfected with stargazin. In the absence of stargazin, ABHD6 overexpression also reduced the amplitude of the currents when any two among GluA1–3 were coexpressed in the HEK293T cells (**Figures 2A–C**). However, similar to GluA1, glutamate elicited almost undetectable ligand-gated currents in HEK293T cells expressing either GluA2 or GluA3 alone (**Figures 2D,E**). We want to address if the existence of endogenous ABHD6 affect the conclusion in **Figures 2D–E**, we used immunostaining and western blotting methods to test the ABHD6 expression level in HEK293T cells (**Supplementary Figure S2**). We found that endogenous ABHD6 can hardly be detected compared with ABHD6-transfected cells, which means that the effect of endogenous ABHD6 in HEK293T cells might be neglected. Furthermore, in the absence of GluAs, the expression of stargazin alone in HEK293T cells exhibited zero current in response to the glutamate puffing (**Figure 2F**), demonstrating the absence of endogenously expressed GluAs in native HEK293T cells. These results showed that the overexpression of ABHD6 inhibits glutamate-induced currents mediated by either heterophilic or hemophilic AMPARs in transfected HEK293T cells.

### The C-Terminus of GluAs Mediated the Inhibitory Effect of ABHD6

**FIGURE 3:**
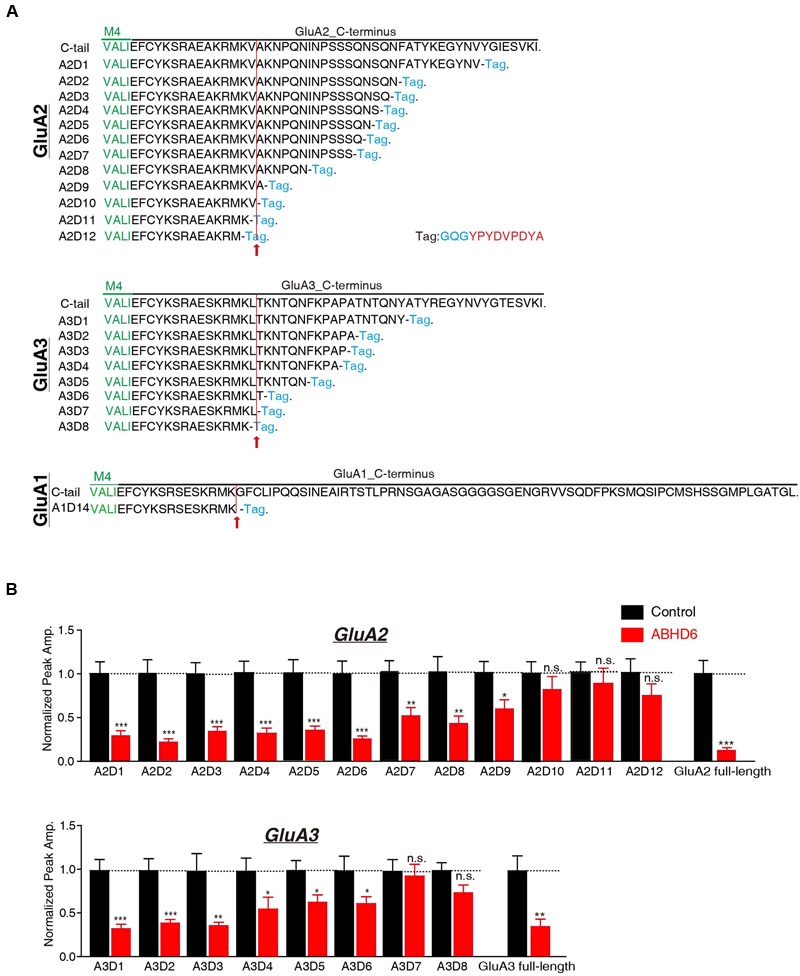
**The C-terminus of GluAs mediated the inhibitory effect of ABHD6. (A)** The amino acid sequences of different GluA1–3 deletion constructs. The arrow points to the mutants after which ABHD6 failed to reduce the amplitude of the glutamate-induced current. **(B)** Summary graphs of the peak amplitudes of 10 mM glutamate-induced currents in HEK293T cells transfected with different GluA2/GluA3 deletions, stargazin, and a control vector or vectors encoding ABHD6 (A2D1, control: 24/3, ABHD6: 24/3, *p* < 0.0001; A2D2, control: 26/3, ABHD6: 26/3, *p* < 0.0001; A2D3, control: 24/3, ABHD6: 24/3, *p* < 0.0001; A2D4, control: 24/3, ABHD6: 24/3, *p* < 0.0001; A2D5, control: 25/3, ABHD6: 24/3, *p* < 0.001; A2D6, control: 27/3, ABHD6: 27/3, *p* < 0.001; A2D7, control: 32/4, ABHD6: 32/4, *p* < 0.01; A2D8, control: 29/3, ABHD6: 29/3, *p* < 0.01; A2D9, control: 34/4, ABHD6: 34/4, *p* < 0.05; A2D10, control: 26/3, ABHD6: 26/3, *p* > 0.05; A2D11, control: 27/3, ABHD6: 27/3, *p* > 0.05; A2D12, control: 26/3, ABHD6: 26/3, *p* > 0.05; GluA2 full-length, 30/3, ABHD6: 30/3, *p* < 0.0001; A3D1, control: 24/3, ABHD6: 24/3, *p* < 0.0001; A3D2, control: 26/3, ABHD6: 26/3, *p* < 0.001; A3D3, control: 25/3, ABHD6: 25/3, *p* < 0.01; A3D4, control: 24/3, ABHD6: 24/3, *p* < 0.05; A3D5, control: 26/3, ABHD6: 26/3, *p* < 0.05; A3D6, control: 25/3, ABHD6: 25/3, *p* < 0.05; A3D7, control: 27/3, ABHD6: 27/3, *p* > 0.05; A3D8, control: 26/3, ABHD6: 26/3, *p* > 0.05; GluA3 full-length, 27/3, ABHD6: 27/3, *p* < 0.01). Each graph of the peak amplitude in HEK293T cells transfected with various ABHD6-expressing vectors was normalized to the peak amplitude in HEK293T cells transfected with the control vector. All summary graphs show means ± SEMs; statistical comparisons by Student’s *t*-test yielded: ^∗^*p* < 0.05, ^∗∗^*p* < 0.01, ^∗∗∗^*p* < 0.001.

The C-terminus of GluA1 has been shown to be crucial for ABHD6’s inhibitory effect in heterologous cells. ABHD6 failed to reduce the glutamate-induced current in HEK293T cells expressing a GluA1 mutant in which the C-tail was deleted after “SKRMK” (Wei et al., 2016). We then investigated the importance of similar C-terminal regions in GluA2 and GluA3 for ABHD6-induced inhibition. To this end, we cloned 12 GluA2 C-terminal deletion constructs and eight GluA3 C-terminal deletion constructs based on the sequence similarity among GluA1, GluA2, and GluA3 (**Figure 3A**). Using full-length GluA2 or GluA3 as a positive control, ABHD6 failed to reduce the amplitude of the glutamate-induced current in HEK293T cells expressing GluA2 mutants (A2D10, A2D11, A2D12) and GluA3 mutants (A3D7 and A3D8)(**Figure 3B** and **Supplementary Figure S1**). Interestingly, these results point out that the longest ABHD6-resistant GluA mutants, A2D10 (ending with AKRMKV in GluA2) and A3D7 (ending with SKRMKL in GluA3), and the previously identified A1D14 (ending with SKRMK in GluA1), share sequence similarity to some extent (**Figure 3A**). Thus, like GluA1, the C-terminal regions of GluA2 and GluA3 are required for ABHD6’s inhibition of AMPAR-mediated currents in transfected HEK293T cells.

**FIGURE 4:**
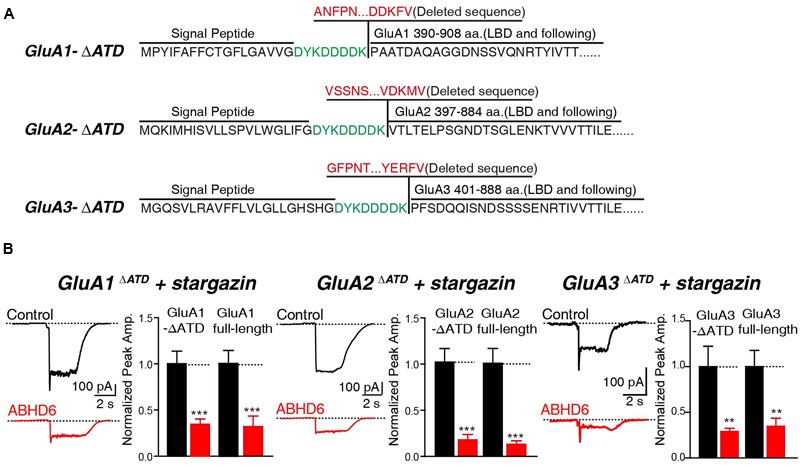
**The N-terminus of GluAs were not required for ABHD6-induced inhibition. (A)** The amino acid sequences of GluA1–3 N-terminal deletion constructs. **(B)** Representative traces (left) and summary graphs of the peak amplitudes (right) of 10 mM glutamate-induced currents in HEK293T cells transfected with GluA1–3 N-terminal deletions, stargazin, and a control vector or vectors encoding ABHD6 (GluA1^ΔATD^, control: 24/3, ABHD6: 24/3, *p* < 0.001; GluA1 full-length, control: 27/3; ABHD6: 27/3; peak: *p* < 0.001; GluA2^ΔATD^, control: 26/3, ABHD6: 26/3, *p* < 0.0001; GluA2 full-length, control: 30/3; ABHD6: 30/3; peak: *p* < 0.0001; GluA3^ΔATD^, control: 24/3, ABHD6: 24/3, *p* < 0.01; GluA3 full-length, control: 27/3; ABHD6: 27/3; peak: *p* < 0.01). Each graph of the peak amplitude in HEK293T cells transfected with various ABHD6-expressing vectors was normalized to the peak amplitude in HEK293T cells transfected with the control vector. All summary graphs show means ± SEMs; statistical comparisons by Student’s *t*-test yielded: ^∗^*p* < 0.05, ^∗∗^*p* < 0.01, ^∗∗∗^*p* < 0.001.

In addition, we generated N-terminal partial deletion mutants for GluA1, GluA2, and GluA3, and tested these mutants in transfected HEK293T cells. Because the extracellular ligand-binding domain is essential for the receptors to respond to glutamate, we removed the entire amino-terminal domain (ATD) from GluA1–3 (**Figure 4A**). The results showed that in cells expressing GluA1ΔATD, GluA2ΔATD, and GluA3ΔATD, ABHD6 suppressed the glutamate-induced currents to a similar extent as those of full-length GluA1–3 (**Figure 4B**). These results demonstrated that the ATDs of GluAs are not required for ABHD6’s inhibition of AMPAR-mediated currents.

### Overexpression of ABHD6 Suppressed the Surface Expression of GluA1–3 in the Transfected HEK293T Cells

**FIGURE 5:**
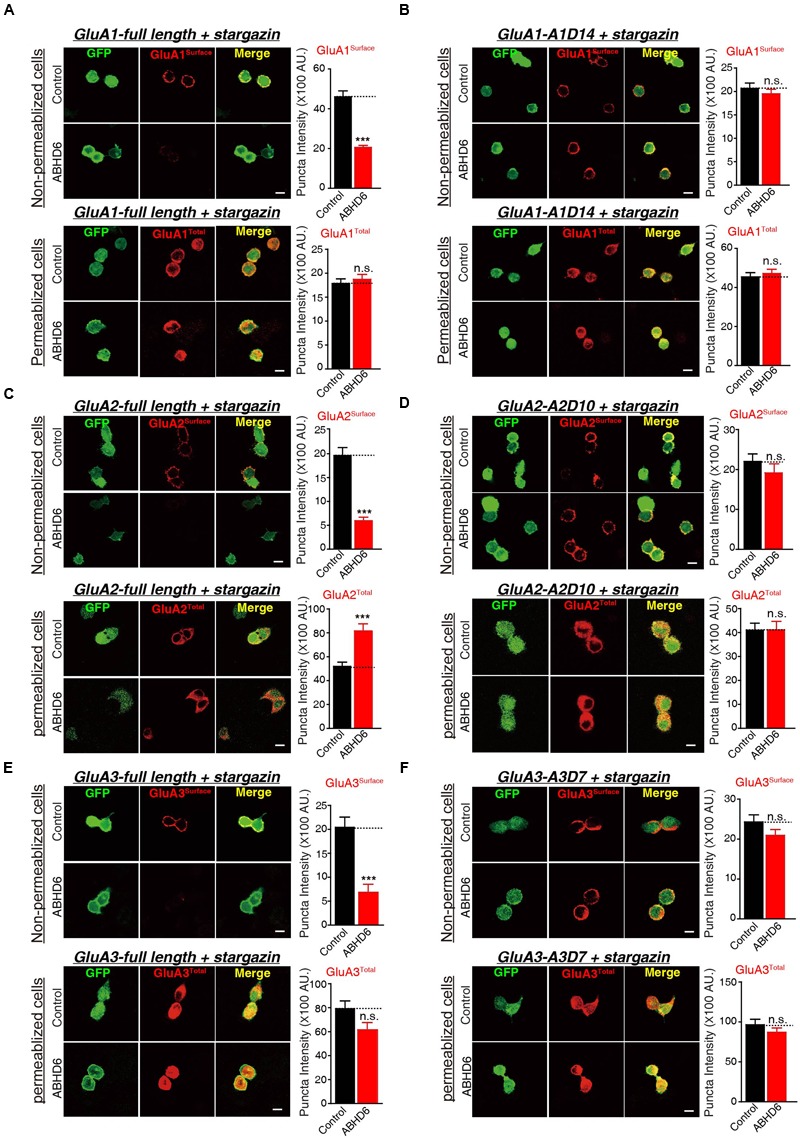
**Overexpression of ABHD6 suppressed the surface expression of GluA1–3 in the transfected HEK293T cells.** The white lines represent the scale bar (scale bar = 10 μm). **(A)** Measurement of the surface (top) and total (bottom) expression of GluA1 in HEK293T cells expressing ABHD6 or a control plasmid together with GluA1 and stargazin. The transfected HEK293T cells were stained with or without permeabilization using an anti-GluA1 antibody. The panels show representative images (left) and quantification of the puncta intensity (right) (control: *n* = 182/3; ABHD6: *n* = 191/3; surface GluA1: *p* < 0.0001; control: *n* = 233/3; ABHD6: *n* = 218/3; total GluA1: *p* > 0.05). **(B)** Measurement of the surface (top) and total (bottom) expression of GluA1 C-terminal deletion A1D14 in HEK293T cells expressing ABHD6 or a control plasmid together with A1D14 and stargazin. The transfected HEK293T cells were stained with or without permeabilization using an anti-GluA1 antibody. The panels show representative images (left) and quantification of the puncta intensity (right) (control: *n* = 248/3; ABHD6: *n* = 208/3; surface GluA1: *p* > 0.05; control: *n* = 152/3; ABHD6: *n* = 154/3; total GluA1: *p* > 0.05). **(C)** Measurement of the surface (top) and total (bottom) expression of HA-tagged GluA2 in HEK293T cells expressing ABHD6 or a control plasmid together with GluA2 and stargazin. The transfected HEK293T cells were stained with or without permeabilization using an anti-HA antibody. The panels show representative images (left) and quantification of the puncta intensity (right) (control: *n* = 169/3; ABHD6: *n* = 187/3; surface GluA2: *p* < 0.0001; control: *n* = 147/3; ABHD6: *n* = 141/3; total GluA2: *p* < 0.0001). **(D)** Measurement of the surface (top) and total (bottom) expression of HA-tagged GluA2 C-terminal deletion A2D10 in HEK293T cells expressing ABHD6 or a control plasmid together with A2D10 and stargazin. The transfected HEK293T cells were stained with or without permeabilization using an anti-HA antibody. The panels show representative images (left) and quantification of the puncta intensity (right) (control: *n* = 97/3; ABHD6: *n* = 103/3; surface GluA2: *p* > 0.05; control: *n* = 115/3; ABHD6: *n* = 111/3; total GluA2: *p* > 0.05). **(E)** Measurement of the surface (top) and total (bottom) expression of HA-tagged GluA3 in HEK293T cells expressing ABHD6 or a control plasmid together with GluA3 and stargazin. The transfected HEK293T cells were stained without or with permeabilization using an anti-HA antibody. The panels show representative images (left) and quantification of the puncta intensity (right) (control: *n* = 139/3; ABHD6: *n* = 123/3; surface GluA3: *p* < 0.0001; control: *n* = 144/3; ABHD6: *n* = 111/3; total GluA3: *p* > 0.05). **(F)** Measurement of the surface (top) and total (bottom) expression of HA-tagged GluA3 C-terminal deletion A3D7 in HEK293T cells expressing ABHD6 or a control plasmid together with A3D7 and stargazin. The transfected HEK293T cells were stained with or without permeabilization using an anti-HA antibody. The panels show representative images (left) and quantification of the puncta intensity (right) (control: *n* = 151/3; ABHD6: *n* = 164/3; surface GluA3: *p* > 0.05; control: *n* = 101/3; ABHD6: *n* = 103/3; total GluA3: *p* > 0.05). All summary graphs show means ± SEMs; statistical comparisons by Student’s *t*-test yielded: ^∗^*p* < 0.05, ^∗∗^*p* < 0.01, ^∗∗∗^*p* < 0.001.

To investigate whether the observed reduction in AMPAR-mediated currents in the heterogonous cells is due to a specific loss of surface-localized AMPARs, or an overall reduction in the expression level of total GluAs, we performed quantitative immunostaining of GluA1–3 from both permeabilized and non-permeabilized HEK293T cells expressing various GluAs together with stargazin. Our results revealed that the coexpression of ABHD6 reduced the surface expression of GluA1 (**Figure 5A**), GluA2 (**Figure 5C**), or GluA3 (**Figure 5E**) compared with the control groups. However, the total GluA1 immunostaining signal from permeabilized HEK293T cells showed no difference between HEK293T cells expressing ABHD6 and stargazin together with GluA1 (**Figure 5A**) or GluA3 (**Figure 5E**), compared to control groups. Furthermore, overexpression ABHD6 increased the total expression of full-length GluA2 (**Figure 5C**). Thus, ABHD6 specifically affected the surface expression of GluA subunits when coexpressed with stargazin.

Because ABHD6 failed to reduce the ligand-induced currents when expressing C-terminal deletion mutants of GluAs (**Figure 3**; GluA2-A2D10, and GluA3-A3D7, and GluA1-A1D14), we next explored whether these mutations are also resistant to the effects of ABHD6 on the surface expression of AMPARs. By quantifying the immunostaining signal from non-permeabilized and permeabilized HEK293T cells expressing A1D14 (**Figure 5B**), A2D10 (**Figure 5D**), and A3D7 (**Figure 5F**), we found that the ABHD6-induced suppression of surface GluAs was abolished, and the total expression levels of GluAs remained unchanged compared to control groups. These results suggested that the same GluAs C-terminal mutants abolished the inhibitory effect of ABHD6 on glutamate-induced currents and the surface expression of AMPARs.

### ABHD6 Directly Bound to GluA1–3 Though the C-Terminus of the Receptors

**FIGURE 6:**
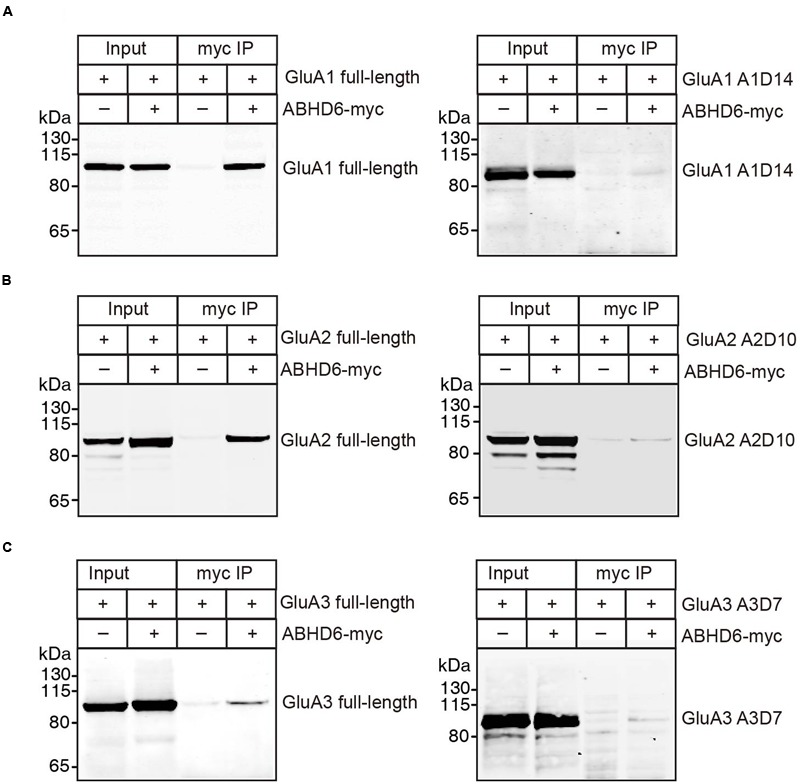
**ABHD6 directly bound to GluA1–3 though the C-terminus of the receptors.** Pull-down of GluA1–3 or various deletions in HEK 293T cells. ABHD6 was immobilized by an myc-tag antibody, and binding was visualized by detection for the GluA1 or HA-tag. **(A)** Pull-down of GluA1 (left) or HA-tagged A1D14 (right) expressed in transfected HEK293T cells together with myc-tagged ABHD6 by an anti-myc antibody. **(B)** Pull-down of HA-tagged GluA2 (left) or A2D10 (right) expressed in transfected HEK293T cells together with myc-tagged ABHD6 by an anti-myc antibody. **(C)** Pull-down of HA-tagged GluA3 (left) or A3D7 (right) expressed in transfected HEK293T cells together with myc-tagged ABHD6 by an anti-myc antibody.

The notion of ABHD6–AMPAR association was first proposed by a high-resolution proteomics study (Schwenk et al., 2012), and later confirmed by our biochemistry study showing that ABHD6 coimmunoprecipitated GluA1 in transfected heterologous cells (Wei et al., 2016). In the present study, we used a similar approach to test whether ABHD6 coimmunoprecipitated GluA2 and GluA3 when transfected in the HEK293T cells. We found that ABHD6 was specifically coimmunoprecipitated with full-length GluA2 (**Figure 6B**) or GluA3 (**Figure 6C**) only when ABHD6 and GluA1 were coexpressed. Next, to test whether the interactions between ABHD6 and GluA2/3 also require the C-tail of the GluA subunits, we used ABHD6 as a bait to coimmunoprecipitate GluA2 and GluA3 C-terminal mutations that abolished ABHD6’s inhibitory effect on the ligand-induced currents and surface expression of GluAs in transfected HEK293T cells. We found that the C-terminal deletion mutations, GluA1-A1D14 (**Figure 6A**), GluA2-A2D10 (**Figure 6B**), and GluA3-A3D7 (**Figure 6C**) all failed to coimmunoprecipitate with ABHD6 in the pull-down assay. These results clearly suggested that ABHD6 binds to all three GluA subunits through their C-terminus domains, and implied that this binding might serve as the underlying mechanism for the functional effects observed in **Figures 1–3**.

## Discussion

ABHD6, a monoacylglycerol lipase, was previously found to inhibit the glutamate-induced currents of GluA1-containing AMPARs in both heterologous cells and neurons (Wei et al., 2016). In this study, we extended our observations to GluA2- and GluA3-containing AMPARs. Our results show that the overexpression of ABHD6 significantly reduced the peak amplitude and steady-state amplitude of glutamate-induced currents in HEK293T cells expressing GluA2, GluA3, or GluA2 + GluA3 when co-transfected with stargazin (**Figure 1**). In the absence of stargazin, ABHD6 overexpression also reduced the amplitude of currents when any two among GluA1–3 were coexpressed in the HEK293T cells (**Figure 2**). Our results also revealed that the suppression effect of ABHD6 on the surface AMPAR levels is independent of the subunit composition of the AMPARs in transfected HEK293T cells (**Figure 5**).

Similar to previous findings in GluA1, the C-terminal domains of GluA2 and GluA3 are required for ABHD6 to inhibit the ligand-gated current in transfected HEK293T cells (**Figure 3**), and to bind with AMPARs in the co-immunoprecipitation (co-IP) experiments. ABHD6 can significantly decrease GluA1–3 surface expression, but without the C-terminus, the GluA1–3 surface expression level was restored (**Figure 5**). Combining this with the co-IP experiments, we can infer that ABHD6 directly binds to GluA1–3 C-terminal regions, and selectively inhibits the surface delivery of AMPARs. These observations also suggest that ABHD6 functions during receptor trafficking. According to our results, ABHD6 can interact with the GluA1–3 C-terminus, but the specific interaction region or motif still needs further investigation. The binding of ABHD6 with the GluA1–3 C-terminus is consistent with the observation that ABHD6 is a membrane-bound protein, but is different from stargazin, which interacts with the glutamate-binding domain of AMPARs (Tomita et al., 2007). Stargazin increased the ligand-induced current in oocytes expressing full-length GluA1 or in GluA1 lacking the ATD. However, the introduction of a Lurcher mutation (A636T) in the glutamate-binding domain of GluA1, or a L497Y mutation in the extracellular S1 domain, abolished the stargazin’s enhancement of glutamate-induced currents (Tomita et al., 2007). Thus, ABHD6 and stargazin interact with different domains of AMPARs.

Furthermore, our data clearly demonstrated that ABHD6’s inhibition on AMPAR trafficking is not dependent on the existence of stargazin. This is in contrast to CNIH-2/3 or PORCN, two other auxiliary proteins recently found in the macro-complex of AMPARs (Mauric et al., 2013). The overexpression of CNIH-2/3 facilitated the membrane delivery of AMPARs and increased the amplitude of glutamate-elicited currents in heterologous cells and neurons (Schwenk et al., 2009; Kato et al., 2010; Shi et al., 2010; Coombs et al., 2012; Gill et al., 2012; Harmel et al., 2012). CNIH-2/3 colocalized with γ-8 but not found in the AMPAR complex lack stargazin (γ-2) and γ-3 (Schwenk et al., 2009; Kato et al., 2010; Gill et al., 2011). The membrane localization of CNIH-2 is critically dependent on γ-8, reflected as the absence of surface CNIH-2 in the cerebellum where γ-8 was also absent (Gill et al., 2011). This effect is subject to TARP modulation, since coexpression of CNIH-2 with GluAs and γ-2 showed a more profound increase in current intensity in heterologous cells than that in γ-8 (Gill et al., 2012). PORCN is another auxiliary protein found in the AMPAR macro-complex (Schwenk et al., 2012). Similar to ABHD6, PORCN negative regulates the trafficking of AMPARs in both transfected heterologous cells and knockout (KO) neurons. However, the effect of PORCN seems to be mediated with a TAPR-dependent mechanism. Knockdown PORCN dissociates γ-8 from the AMPAR complex and alters the subunit composition of AMPARs in the hippocampal neurons (Erlenhardt et al., 2016). Thus, ABHD6 and stargazin function in a divergent manner in trafficking the AMPARs to the plasma membrane.

## Conclusion

We extend our previous observation on the functional interaction of ABHD6 and GluA1-containing receptors to GluA2- and GluA3-containing receptors. These results revealed a negative mechanism governing the membrane trafficking of AMPARs through ABHD6 that is independent on stargazin. The limitation of these studies is the lack of *in vivo* studies of this interaction using KO animals, which requires further investigation.

## Author Contributions

MW, MJ, and JZ contributed equally to this work. WZ, YS, and CZ designed research; MW, MJ, and JZ performed research and analyzed data; LY, YZ, YC, and YM analyzed data; WZ, YS, and CZ wrote the paper.

## Conflict of Interest Statement

The authors declare that the research was conducted in the absence of any commercial or financial relationships that could be construed as a potential conflict of interest.

The reviewer GC declared a shared affiliation with one of the authors YS to the handling Editor, who ensured that the process nevertheless met the standards of a fair and objective review.

## Supplementary Material

FIGURE S1 **The C-terminus of GluAs mediated the inhibitory effect of ABHD6. (A)** Summary graphs of the peak amplitudes of 10 mM glutamate-induced currents in HEK293T cells transfected with GluA2 deletion A2D10, A2D11, A2D12, and GluA2 full-length. **(B)** Summary graphs of the peak amplitudes of 10 mM glutamate-induced currents in HEK293T cells transfected with GluA3 deletion A3D7, A3D8, and GluA3 full-length. (A2D10, control: 26/3, ABHD6: 26/3, *p* > 0.05; A2D11, control: 27/3, ABHD6: 27/3, *p* > 0.05; A2D12, control: 26/3, ABHD6: 26/3, *p* > 0.05; GluA2 full-length, 30/3, ABHD6: 30/3, *p* < 0.0001; A3D7, control: 27/3, ABHD6: 27/3, *p* > 0.05; A3D8, control: 26/3, ABHD6: 26/3, *p* > 0.05; GluA3 full-length, 27/3, ABHD6: 27/3, *p* < 0.01).

FIGURE S2 **The expression level of endogenous ABHD6 in HEK293T cells is very low compared with HEK293T cells transfected with ABHD6.** The white lines represent the scale bar (scale bar 10μm). **(A)** Measurement of the expression level of ABHD6 in HEK293T cells expressing ABHD6 (bottom) or a control plasmid (top). The transfected or untransfected HEK293T cells were stained with permeabilization using an anti-ABHD6 antibody. The panels show representative images. **(B)** Measurement of the expression level of ABHD6 in HEK293T cells expressing ABHD6 (right) or a control plasmid (left) using Western blotting. ABHD6 was detected using an anti-ABHD6 antibody.
