# Supplementary figures and images for: The Inhibitory Effect of α/β-Hydrolase Domain-Containing 6 (ABHD6) on the Surface Targeting of GluA2- and GluA3-Containing AMPA Receptors
Source: Front Mol Neurosci. 2017 Mar 2;10:55. doi: 10.3389/fnmol.2017.00055 (PMC5333494; doi:10.3389/fnmol.2017.00055)

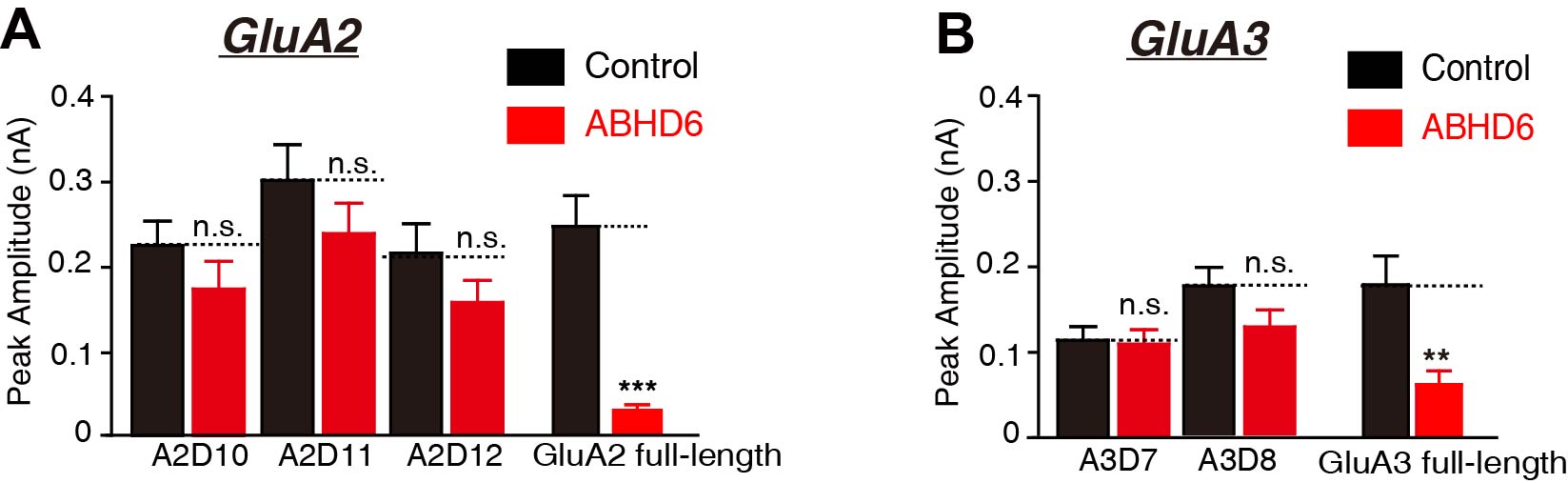

Supplement: FIGURE S1 — The C-terminus of GluAs mediated the inhibitory effect of ABHD6. (A) Summary graphs of the peak amplitudes of 10 mM glutamate-induced currents in HEK293T cells transfected with GluA2 deletion A2D10, A2D11, A2D12, and GluA2 full-length. (B) Summary graphs of the peak amplitudes of 10 mM glutamate-induced currents in HEK293T cells transfected with GluA3 deletion A3D7, A3D8, and GluA3 full-length. (A2D10, control: 26/3, ABHD6: 26/3, p > 0.05; A2D11, control: 27/3, ABHD6: 27/3, p > 0.05; A2D12, control: 26/3, ABHD6: 26/3, p > 0.05; GluA2 full-length, 30/3, ABHD6: 30/3, p < 0.0001; A3D7, control: 27/3, ABHD6: 27/3, p > 0.05; A3D8, control: 26/3, ABHD6: 26/3, p > 0.05; GluA3 full-length, 27/3, ABHD6: 27/3, p < 0.01). [file Image_1.JPEG]

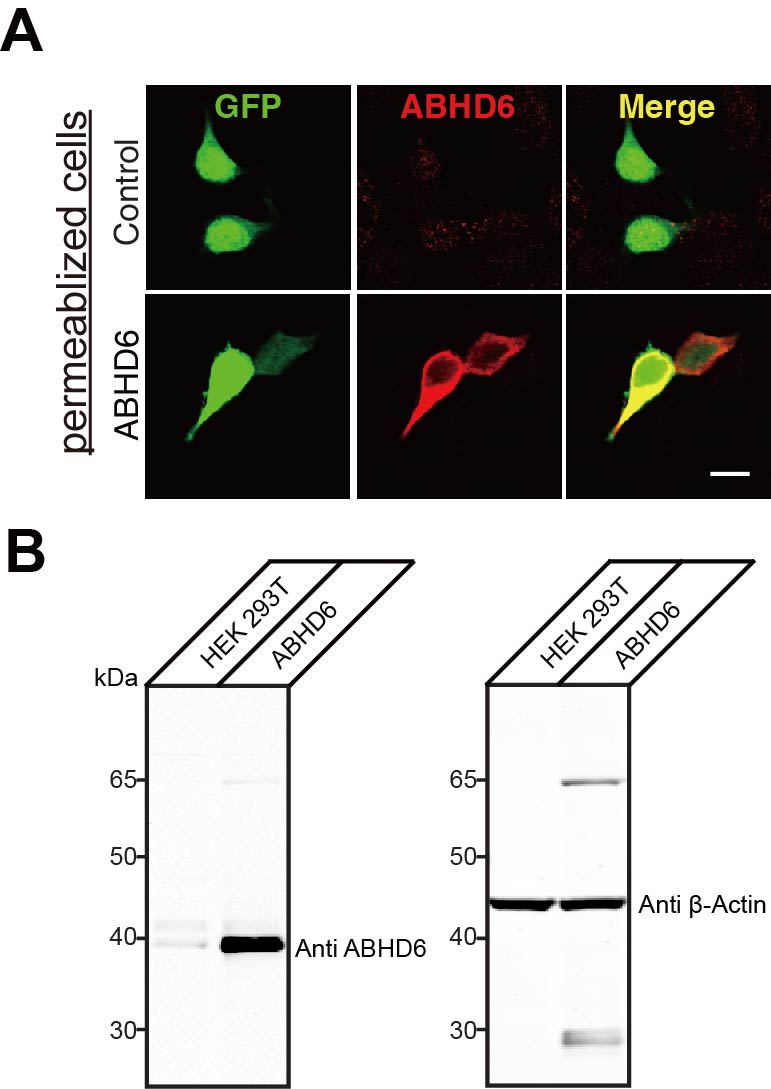

Supplement: FIGURE S2 — The expression level of endogenous ABHD6 in HEK293T cells is very low compared with HEK293T cells transfected with ABHD6. The white lines represent the scale bar (scale bar 10μm). (A) Measurement of the expression level of ABHD6 in HEK293T cells expressing ABHD6 (bottom) or a control plasmid (top). The transfected or untransfected HEK293T cells were stained with permeabilization using an anti-ABHD6 antibody. The panels show representative images. (B) Measurement of the expression level of ABHD6 in HEK293T cells expressing ABHD6 (right) or a control plasmid (left) using Western blotting. ABHD6 was detected using an anti-ABHD6 antibody. [file Image_2.JPEG]
